# Treatment of a Prader-Willi Patient with Recurrent Catatonia

**DOI:** 10.1155/2015/697428

**Published:** 2015-05-07

**Authors:** Hana M. Poser, Alexandru E. Trutia

**Affiliations:** ^1^Medical College of Virginia, 1201 E. Marshall Street, Richmond, VA 23284, USA; ^2^Department of Psychiatry, Virginia Commonwealth University Health Systems, 1300 W. Broad Street, Richmond, VA 23284, USA

## Abstract

Prader-Willi is a genetic disorder characterized by neonatal hypotonia, hyperphagia, short stature, hypogonadism, and mental delay. This disorder can result from multiple mechanisms, most commonly a deletion of paternal chromosome 15, leaving a single maternally derived chromosome 15. Individuals who have a maternal uniparental disomy of chromosome 15 have a higher risk for developing psychosis compared to other forms of Prader-Willi. The following report details the treatment course of a 24-year-old female with Prader-Willi and recurrent catatonia. The patient initially had a positive lorazepam challenge test but subsequently failed treatment with benzodiazepines. She then received eight electroconvulsive therapy (ECT) treatments after which she showed improvement from initial catatonic state. However, the resolution in her symptoms did not follow a linear course but would show periods of improvement followed by a return of catatonic features. This case provides an example of the complexity of treatment of a patient with a genetic disorder and recurrent catatonia.

## 1. Introduction

Prader-Willi syndrome (PWS) is a genetic disorder with an incidence of one in 29,000 [1] characterized by absence of expression of one or more paternally inherited genes on chromosome 15q11-q13. Those with PWS have a characteristic phenotype which includes neonatal hypotonia, hypogonadism, short stature, decreased cognition, and most classically extreme overeating that can lead to obesity without food restriction. There are four genetic mechanisms which can result in the Prader-Willi genotype. Approximately 70% of individuals affected have a de novo deletion of the paternal chromosome 15 (15q11-q13), 25% of individuals have a maternal uniparental disomy (mUPD) with two copies of maternal chromosome 15, and the remaining 5% result from an imprinting defect or unbalanced chromosomal translocation [2]. Previous studies have shown that those individuals with the mUPD are at a much higher risk for psychosis than those with PWS due to some other genetic causes [3, 4]. In addition, individuals with the mUPD had a greater incidence of recurrent psychiatric episodes and often those with recurrences showed deterioration from premorbid functioning [4]. The limited studies on PWS consist of case reports and cohort studies with relatively small sample sizes due to the low prevalence of this syndrome. One case report exists describing a 17-year-old male with Prader-Willi who presented with catatonia that was responsive to pharmacological therapy alone [5]. The goal of this case report is to detail the clinical course and treatment of catatonia in a patient with PWS of the mUPD type.

## 2. Case Report

Ms. Z is a 25-year-old Caucasian female with Prader-Willi mUPD type, mild mental retardation (IQ 70), and unspecified mood disorder who presented to the emergency department after her parents reported one month of increasingly manic behavior and a ten-pound weight loss in ten days. The patient's behavior five to six days before hospital admission was described as hyperverbal, euphoric, and illogical with decreased sleep. She then suddenly became mute and withdrawn exhibiting purposeless movements prompting her parents to bring her to the hospital.

Ms. Z was diagnosed clinically with Prader-Willi at a young age with the key features of hyperphagia, short stature, hypogonadism, and mental delay all present. It was not until the age of 17 that she underwent genetic testing revealing mUPD form of Prader-Willi. In the past, Ms. Z had two prior hospitalizations at age of 15 and 16 for depression with catatonia that responded to therapy with lorazepam and haloperidol. Before her first hospitalization, Ms. Z had no significant psychological history. Since her first hospitalization, she had been maintained on a variety of antipsychotic and antidepressant regimes for varying diagnosis of mood disorder not otherwise specified (ICD-10 F39), schizoaffective disorder (ICD-10 F25.9), and depression with psychosis (ICD-10 F33.3) while being followed regularly by a pediatric psychiatrist. Her current home regimen on presentation consisted of aripiprazole 25 mg daily, lamotrigine 200 mg daily, and lithium 600 mg two times a day that the patient was compliant with. At baseline, Ms. Z is able to work part-time with children under supervision at a local gym and is described by her parents as very friendly and happy. She lives with her parents who are very involved in her care.

At initial exam, Ms. Z appeared a well-groomed, obese young female with stooped posture and mild hand tremor present bilaterally. She appeared drowsy, lethargic, was tearful at times, and did not make eye contact but was able to follow simple commands. She displayed mutism, posturing, waxy flexibility, purposeless movements, repeated shoulder shrugging, and stereotypy with finger pointing, among other behaviors. Bush-Francis Catatonia Rating Scale score totaled 21/23. Her affect was blunted and she was orientated to person but not place or time. Other portions of the exam were not able to be evaluated as patient was unresponsive to questioning. Physical exam was otherwise unremarkable.

During the interview, Ms. Z responded to 1 mg lorazepam challenge with improved verbal responses although speech was very slow, soft, and words were slurred. Head CT did not reveal any acute intracranial abnormality and there was no evidence of seizure on EEG. She was diagnosed with catatonia with depressed features and admitted to the inpatient psychiatric unit where she was started on treatment with lorazepam 1 mg three times a day as well as her home regimen of antipsychotics.

Over the next 10 days Ms. Z showed minimal improvement with lorazepam which was increased to 1 mg every 6 hours. Her symptoms would fluctuate minimally; at times she would be more interactive with staff and family and then return to being withdrawn. During this time, her home regimen was adjusted by decreasing the aripiprazole and lithium and initiating therapy with ziprasidone to help with mood and psychosis. Due to lack of response to pharmacological therapy and concern for patient's well-being with weight loss and dehydration noted on initial presentation, the decision was made to begin ECT therapy with a goal of 6–12 treatments delivered every other day. Ms. Z's parents were supportive of this decision and provided consent for her treatment.

Lithium was discontinued before the start of ECT (refer to Table 1 for treatment details) and her morning dose of lorazepam was held on ECT days. After the first ECT treatment, Ms. Z had improved mental status for about 30 minutes and then returned to initial catatonic state. Following her second treatment with ECT, Ms. Z displayed increased eye contact, was more verbally responsive, had more energy, described her mood as “happier,” and showed decreased psychomotor slowing. During her third ECT session, two stimuli were given due to poor initial motor and EEG seizure responses. The following day, Ms. Z appeared more withdrawn, appeared less interactive with staff, and again appeared to be responding to internal stimuli. Lamotrigine was decreased and held nights before ECT treatment to achieve better ECT response. Lorazepam was also decreased to improve motor response to ECT.

After ECT 4, the patient's progress halted. She displayed increased psychomotor slowing that was very apparent and Ms. Z became very sedated and some of her presenting features of catatonia such as waxy flexibility returned. She was spending more time talking to herself and remained awake for most of the night pacing the hallway. After this notable change, lorazepam was increased due to concern that the lower dosage was responsible for a return back towards catatonic state. Ziprasidone was also increased to help with psychosis.

The following morning, Ms. Z became even more sedated and drowsy, sleeping almost the entire day. Her morning lorazepam was held due to concern over increased sedation and in the following day was again decreased. After ECT 5, she was talkative, interactive, and more energetic. She was able to participate in group activities and took part in dancing, one of her favorite past times. Mini-mental status exam was performed and the patient received 25/30 only missing points for day of the week and date. Her father reported at this point that she was “close to baseline.”

With this improvement, lorazepam was further decreased. ECT 6 was then performed. The night following ECT 6, Ms. Z was noted to be extremely sedated and difficult to arouse. The next morning she provided minimal responses to questioning, was seen smiling to herself most of the day, displayed unsteady gait, increased psychomotor retardation, and was again very drowsy. She did not have any rigidity or purposeless movements. It was unclear whether Ms. Z was moving back towards her catatonic state or if this was just a normal fluctuation of her catatonic state in response to ECT treatment. Lorazepam was discontinued at this time.

ECT 7 was given and over the next three days Ms. Z continued to show psychomotor retardation and was mostly mute but began to engage more on the fourth day. Lastly ECT 8 was given at 100% bitemporal energy due to decreased seizure and waxing/waning mutism displayed at the time. Following her final treatment, Ms. Z still displayed some psychomotor retardation and decreased energy but did have spontaneous speech. Her parents felt she was about 70% of her baseline. The following day, she was able to be discharged home after 29 days of inpatient treatment. She was discharged on lamotrigine 150 mg daily, lithium 300 mg three times a day, and ziprasidone 120 mg daily. At her tenth week of follow-up, Ms. Z's mood had improved. She was able to return to work although she was experiencing some episodes of cataplexy. Six months after hospitalization, she had made a full recovery with no return of catatonic features.

## 3. Discussion

Catatonia is a recognized syndrome characterized by its unique set of behavioral abnormalities and response to benzodiazepines and ECT. Multiple models have been proposed to explain the etiology of these behaviors including theories on dysfunctional GABA receptors and alterations to hypothalamic-pituitary axis [6]. Some also suspect that there could be a genetic component to the development of catatonia. Catatonia can be one of the displayed forms of psychosis seen in individuals with Prader-Willi which is caused by an abnormality in chromosome 15. Similar genes have been implicated in catatonic schizophrenia and autism [6].

Due to the lack of randomized controlled trials addressing the treatment of catatonia, protocol for therapy is mainly based on case reports and retrospective studies. Currently, benzodiazepines followed by ECT or simultaneous use of both represents the standard of care. Case series suggest that benzodiazepines alone have a response rate of 60%–80% [7]. For patients with severe catatonia or catatonia refractory to treatment with benzodiazepines, ECT is the recommended form of therapy. There are no current ECT guidelines for duration of treatment, frequency, strength of treatment, or electrode placement in patients with catatonia due to lack of controlled studies using ECT in this target population. One recent study of 63 patients treated with ECT for catatonia found the average number of ECT sessions per patient to be 7.25 ± 2.54 with an 88.89% response rate [8]. Variations in electrical charge delivered and duration of motor and EEG response can been seen between retrospective studies.

As seen in this case, the treatment course becomes complex when dealing with catatonic patients with comorbid genetic and mood disorders being treated with mood stabilizers and antipsychotics. It becomes difficult to discern what aspects of treatment are contributing to improvement and those that may be hindering it. In addition, the response to ECT can be highly variable among patients and symptoms can seem to fluctuate throughout the course of treatment as seen with Ms. Z. The study by Raveendranathan et al. suggests that characteristics such as younger age, duration of catatonia, and higher Bush Francis may predict better response to ECT. One consistent finding among studies was that earlier diagnosis and treatment of catatonia led to better outcomes. This stresses the importance of early recognition of symptoms, diagnosis, and treatment of catatonia.

A database search locates one other case report of catatonia in a patient with Prader-Willi. This case study described the treatment of a 17-year-old male with Prader-Willi, who was diagnosed clinically and had acute onset catatonia which responded to treatment with lorazepam and risperidone over a two-week period [5]. Ms. Z who was described here with Prader-Willi of the maternal uniparental disomy type was known to have a preexisting mood disorder and received treatment for recurrent catatonia refractory to treatment with benzodiazepines.

## Figures and Tables

**Table 1:** ECT treatment course.

ECT	Medications	Energy/charge	Motor/EEG response	Clinical response
1	Ziprasidone 60 mg twice daily, lamotrigine 200 mg before bed, and lorazepam 1 mg three times daily	25% bifrontal 128.9 mc	1 sec motor/36 sec EEG	Improved verbal response for 30 min and then back to catatonic state. Throughout the day, patient became more verbal and coherent and even expressed humor.

2	Identical regimen as time point 1	40% bifrontal 204.2 mc	10 sec motor/40 sec EEG	Less interactive, disoriented, with repetitive movements.

3	Identical regimen as time point 1	50%/75% bifrontal^1^ 258.7 mc	1 sec motor/7 sec EEG 5 sec motor/48 sec EEG	Less energetic, increasingly drowsy, minimal interaction, decreased sleep, and pacing hallways.

4	Ziprasidone 60 mg twice daily, lamotrigine 150 mg before bed, and lorazepam 0.5 mg three times daily	75% bifrontal 377.4 mc	33 sec motor/56 sec EEG	Very drowsy, psychomotor retardation, decreased sleep, waxy flexibility, and increased speech latency.

5	Ziprasidone 80 mg twice daily, lamotrigine 150 mg before bed, and lorazepam 0.5 mg three times daily	75% bifrontal 376.1 mc	21 sec motor/29 sec EEG	Much more engaged, aware, conversational, and oriented to situation.

6	Ziprasidone 80 mg twice a day, lamotrigine 150 mg before bed (held before ECT), and lorazepam 0.5 twice daily	100% bifrontal 502.4 mc	19 sec motor/34 sec EEG	Waxing/waning mutism, repetitive movements, and psychomotor retardation.

7	Ziprasidone 80 mg twice daily, lamotrigine 150 mg before bed (held before ECT), and d/c lorazepam	100% bifrontal 503.2 mc	19 sec motor/34 sec EEG	Improved but still with low energy and very drowsy.

8	Ziprasidone 40 mg am/80 mg pm, lamotrigine 150 mg before bed	100% bitemporal 502.9 mc	11 sec motor/56 sec EEG	Regaining energy, more engaged, with improved affect.

Morning dose of lorazepam was held in mornings of ECT for better seizure response.

^1^Treatment required two stimuli due to poor motor response.
